# Brain Processing of Contagious Itch in Patients with Atopic Dermatitis

**DOI:** 10.3389/fpsyg.2017.01267

**Published:** 2017-07-25

**Authors:** Christina Schut, Hideki Mochizuki, Shoshana K. Grossman, Andrew C. Lin, Christopher J. Conklin, Feroze B. Mohamed, Uwe Gieler, Joerg Kupfer, Gil Yosipovitch

**Affiliations:** ^1^Institute of Medical Psychology, Justus-Liebig-University Giessen, Germany; ^2^Department of Dermatology, Lewis Katz School of Medicine at Temple University, Philadelphia PA, United States; ^3^Department of Dermatology and Cutaneous Surgery and Miami Itch Center, Miller School of Medicine, University of Miami, Miami FL, United States; ^4^Department of Radiology, Jefferson Integrated Magnetic Resonance Imaging Center, Thomas Jefferson University, Philadelphia PA, United States; ^5^Department of Dermatology, University Hospital of Giessen and Marburg Giessen, Germany

**Keywords:** contagious itch, atopic dermatitis, functional MRI, supplementary motor area, striatum, orbitofrontal cortex

## Abstract

Several studies show that itch and scratching cannot only be induced by pruritogens like histamine or cowhage, but also by the presentation of certain (audio-) visual stimuli like pictures on crawling insects or videos showing other people scratching. This phenomenon is coined “Contagious itch” (CI). Due to the fact that CI is more profound in patients with the chronic itchy skin disease atopic dermatitis (AD), we believe that it is highly relevant to study brain processing of CI in this group. Knowledge on brain areas involved in CI in AD-patients can provide us with useful hints regarding non-invasive treatments that AD-patients could profit from when they are confronted with itch-inducing situations in daily life. Therefore, this study investigated the brain processing of CI in AD-patients. 11 AD-patients underwent fMRI scans during the presentation of an itch inducing experimental video (EV) and a non-itch inducing control video (CV). Perfusion based brain activity was measured using arterial spin labeling functional MRI. As expected, the EV compared to the CV led to an increase in itch and scratching (*p* < 0.05). CI led to a significant increase in brain activity in the supplementary motor area, left ventral striatum and right orbitofrontal cortex (threshold: *p* < 0.001; cluster size *k* > 50). Moreover, itch induced by watching the EV was by trend correlated with activity in memory-related regions including the temporal cortex and the (pre-) cuneus as well as the posterior operculum, a brain region involved in itch processing (threshold: *p* < 0.005; cluster size *k* > 50). These findings suggest that the fronto-striatal circuit, which is associated with the desire to scratch, might be a target region for non-invasive treatments in AD patients.

## Introduction

Itch is regarded as unpleasant and provokes the desire to scratch (Savin, 1998). Many patients with skin diseases suffer from this symptom on a regular basis (Ständer, 2008). Atopic dermatitis (AD) is a chronically relapsing skin disease highly associated with chronic itch (Hanifin and Rajka, 1980). In AD patients, itch often leads to sleeping problems (Lavery et al., 2016) and is associated with a lower psychological wellbeing (Chrostowska-Plak et al., 2013).

In order to better understand physiological processes of itch, methods to induce itch in experimental settings are needed. Two methods that are often used to induce itch in laboratory settings are the application of cowhage (mucuna pruriens) through rubbing or of histamine through iontophoresis (e.g., Papoiu et al., 2011a). However, also the presentation of certain (audio-) visual stimulus material has repeatedly been shown to induce itch. By now there are several studies, which found that the presentation of itch-related stimuli, like pictures of crawling insects or videos showing other people scratching, can trigger itch and scratch responses in both healthy subjects and patients with chronic itch (Niemeier et al., 2000; Papoiu et al., 2011b; Lloyd et al., 2013; Schut et al., 2014, 2015b). However, so far there are only two studies, which investigated brain activity induced by such itch related stimuli (Holle et al., 2012; Mochizuki et al., 2013). The phenomenon that one also feels itch and starts scratching when one is confronted with itch stimuli has been referred to as contagious itch (CI) (Papoiu et al., 2011b; Schut et al., 2015a). CI is similar to another universal phenomenon called contagious yawning (Platek et al., 2005). Interestingly, in psychophysical studies CI and the subsequent scratching behavior were found to be more profound in patients suffering from chronic itch due to AD than in healthy controls (Papoiu et al., 2011b; Schut et al., 2014): Papoiu et al. (2011b) showed that the simultaneous application of a mock stimulus (saline) and an experimental video (EV) showing people scratching led to a significant increase of itch intensity in AD patients, while it only led to a slight increase in itch perception in healthy controls. In accordance, also Schut et al. (2014) found that AD patients displayed a more profound response to audio-visual itch stimuli compared to healthy controls. The patients reported a higher itch increase and displayed more scratching in response to a video on itch-related stimuli than healthy controls. Thus, it has repeatedly been shown that AD patients are more susceptible to (audio-) visual itch-cues than healthy controls. This finding can in terms of associative learning processes possibly be explained by the fact that an association between itch-related stimuli and itch/scratching has been experienced more often by chronic itch patients than healthy controls.

In order to help AD patients it is thus crucial to find ways to break the sequence of itch and scratching resulting in further inflammation known as the itch-scratch cycle (Yosipovitch and Papoiu, 2008) which occurs during CI. Until now, brain processing of CI has only been investigated in healthy controls (Holle et al., 2012; Mochizuki et al., 2013): In the first study, it was found that the presentation of videos showing other people scratching led to an activation of the anterior insular cortex (aIC), the parietal cortex including the primary somatosensory cortex, and the prefrontal cortex (Holle et al., 2012). In another study, an activation of motor-related areas, e.g., the supplementary motor area (SMA) and basal ganglia as well as of parts of the insular cortex were observed when participants were shown itch- related pictures (Mochizuki et al., 2013). Thus, in healthy participants CI goes along with an activation of brain areas that are also activated during itch provoked by pruritogens (Mochizuki et al., 2014). However, the brain processing of CI has never been investigated in AD patients. The investigation of brain processing in this group of patients is of high interest in order to identify brain regions which could be target regions for non-invasive treatments (Mochizuki et al., 2017).

## Materials and Methods

### Subjects

Eleven participants were enrolled in the study: six female and five male AD-patients (mean age: 32.8 ± 11.8 years). All had to have an itch rating of at least 4 out of 10 in a visual analog scale during the past 2 weeks prior to study initiation. All participants were right handed, neurologically healthy and in general good health with no other skin disease, disease state, or physical condition which would impair the evaluation of their itch intensity or which would increase their health risk by study participation. Moreover, all patients were susceptible to the visual itch cues used in our study. In order to verify this, they were presented two videos during a screening visit. Only patients reporting an increase in itch intensity of at least 3 on a visual analog scale ranging from 0 to 10 (0: no itch; 10: worst imaginable itch) due to the EV in comparison to the control video (CV) were included in the study (see also “itch induction”). This inclusion criterion was applied in order to ascertain that we would be able to identify brain regions activated during CI in all participants. Thirteen out of 19 screened subjects, reported an itch increase of at least 3 on a VAS from 0 to 10. Two of the eligible patients did not undergo the fMRI tests due to technical issues and therefore 11 atopics completed the study.

### Ethics Statement

All subjects signed an informed consent before study participation and were free to withdraw from the study at any time. Before the beginning of the study, the study protocol had been approved by the Institutional Review Board (IRB) of the Temple University School of Medicine, Philadelphia, PA, United States and was found to conform to the guidelines of the World Health Organization (Declaration of Helsinki).

### Design and Procedure

While lying in the scanner, participants were presented with two videos, one of which induced itch (EV) and a second one, which was not itch-inducing (CV). The order of video presentation varied between participants. Before each video presentation, the patients were instructed to keep their head still and to relax as much as possible during the video presentation. Participants were told that they were not allowed to scratch in the scanner. After each video presentation, they were asked to report their itch ratings using a response button. In between the videos, there was a 30 min wash-out period. This was in particular important in order to ensure that the itch due to the EV had fully disappeared during the presentation of the CV. During the wash-out period, all participants remained inside the scanner and were told that they were not allowed to fall asleep.

The same procedure of itch induction was repeated outside the scanner after the MRI measurements. This was done in order to be able to also measure the scratching behavior outside the scanner during CI.

### Itch Induction

Itch was induced by a video showing people scratching (EV). A video showing the same people sitting idle was used as a CV. Both videos lasted 5 min and 50 s. The videos consisted of ten sequences, which lasted 30 s each. In addition, in between these sequences a pause of 5 s was included. The 30 s sequences each showed one person, who either scratched the right forearm (EV) or sat idle looking straightforward (CV). The persons appeared in the same order in both videos. Two of the shown persons were female and one was male. These videos were validated in a previous study, in which it was shown that they led to an increase in itch/scratching in AD-patients and healthy controls (Papoiu et al., 2011b).

### Psychophysical Measurements

The average itch intensity that occurred due to the videos was measured by means of a visual analog scale ranging from 0 to 10 (0: no itch, 10: worst itch imaginable) immediately after each video presentation. The patients were asked to answer the following question using a response button in the scanner: “How itchy have you felt during the last video presentation on average?”. Moreover, the number of scratch movements and the scratch duration that occurred during the presentations of the videos outside the scanner were video-recorded and subsequently rated by two independent persons not involved in data collection, who did not know the sequence of video presentation. The interrater-reliability was *r* ≥ 0.90 for all videos.

### MRI Measurement and Data Analysis

Due to the fact that participants in this study continuously viewed the videos for 5:50 min to induce itch, which can induce slow changes of neural activity in the brain, we decided to use arterial spin labeled (ASL)-functional MRI (fMRI) to measure brain activity, because this method is suitable for detecting such slow changes. The scanner that was used was a 3Tesla Verio MR scanner, Siemens, Erlangen, Germany. The Pseudo-Continuous Arterial Spin Labeling (pCASL) pulse consisted of 1520 selective radiofrequency (RF) pulses (Hanning window, B1 average = 1.8 μT, duration = 500 μs, spacing = 500 μs, *G*_average_ = 1 mT/m, *G*_maximum_/*G*_average_ = 8) with a labeling duration of 1527.6 ms and post-labeling delay (PLD) of 1500 ms. *G*_maximum_ represents the gradient amplitude during each Hanning pulse. *G*_average_ represents the average gradient applied between the center of two RF pulses. For the control, the RF phase alternated from 0 to 180°. Identical G waveforms were used for label and control acquisitions. The inversion plane was offset 8 cm from the center of the field-of-view (FOV) in the head–foot direction, so that it was located at the base of the cerebellum to achieve good labeling efficiency. The readout parameters were selected as follows: TR = 4000 ms, echo time (TE) = 12 ms, excitation flip angle = 90°, in-plane resolution = 3.4 mm × 3.4 mm, matrix = 64 × 64, slice thickness = 6 mm, 16 slices with 3 mm gap acquired in ascending order, BW = 2790 Hz/px, FOV = 256 × 256 mm2, phase-encoding direction = anterior–posterior (A–P), total readout time (for all 16 slices) = 700 ms.

We used statistical parametric mapping 8 (SPM8^1^) for analysis of the MRI data. Cerebral blood flow (CBF) image was obtained by processing the MRI data using the published toolbox to create CBF image (ASLtbx^2^). Then, we subtracted the CBF image in the CV condition from that in the EV condition. This subtracted image (i.e., delta-CBF image) was then spatially normalized to MNI template brain and smoothed with a Gaussian kernel (6.4 mm × 6.4 mm × 9 mm) using SPM. In order to detect brain areas that were activated during CI, a one sample *t*-test was conducted. In addition, we also performed correlation analysis using delta-CBF image and difference in itch intensity between CV and EV (i.e., delta-itch intensity). In this analysis, we investigated brain regions in that activity during EV showed significant correlation with delta-itch intensity. Analysis of co-variance (ANCOVA) was applied in the above statistical tests to minimize global effects (i.e., individual difference in global delta-CBF). Voxel-level threshold for above SPM analyses was set at uncorrected *p* < 0.001. Cluster-level threshold for the analyses was set at greater than 50 for minimizing a risk to detect artifacts as significantly activated areas.

### Analysis of Psychophysical Data

**FIGURE 1:**
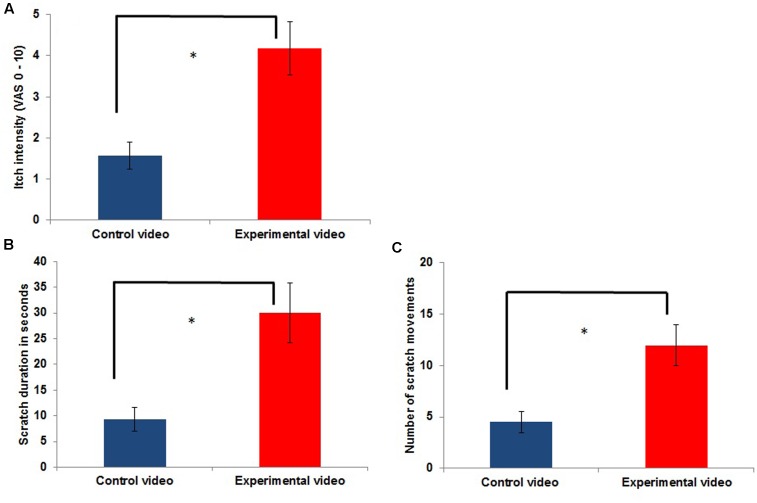
Itch intensity **(A)** and scratching **(B,C)** *outside the scanner* during the control condition [presentation of a control video (CV)] compared to itch during the experimental condition [contagious itch (CI) condition: presentation of a video showing other people scratching; experimental video (EV); *n* = 11; ^∗^*p* < 0.05].

**FIGURE 2:**
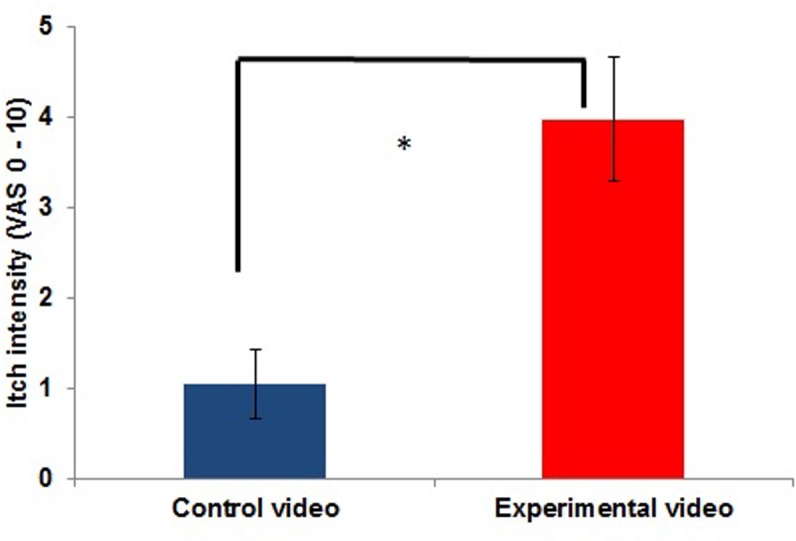
Itch intensity *inside the scanner* during the control condition (presentation of a CV) compared to itch during the experimental condition (CI condition: presentation of a video showing other people scratching; EV; *n* = 11; ^∗^*p* < 0.05).

The statistical analysis of the psychophysical data was performed using SPSS 22 (IBM, Ehningen, Germany). Paired-samples *t*-tests were used to determine whether the average itch intensity and scratching significantly differed during the presentation of the EV and CV. The intended level of significance was *p* < 0.05. Means ± SEM are presented in **Figures 1**, **2**.

## Results

### Itch Induction and Scratching Behavior (Outside of the Scanner)

As shown in **Figure 1**, itch intensity was significantly higher during an itch inducing EV (EV condition) compared to the presentation of the CV (CV condition) [*t*(10) = -4.980; *p* = 0.001]. Frequency and duration of scratching in the EV condition were also significantly increased compared to those in the CV condition (frequency of scratching: [*t*(10) = -4.366; *p* = 0.001]; duration of scratching: [*t*(10) = -3.528; *p* = 0.009]; **Figure 1**). The mean itch intensity during the presentation of the CV was 1.57 ± 1.11, while it was 4.18 ± 2.14 during the presentation of the EV. The mean scratch duration and number of scratch movements during the presentation of the CV was 9.27 ± 7.68 s and 4.5 ± 3.35 scratch movements, respectively, while it was 30 ± 19.49 s and 11.95 ± 6.64 scratch movements during the presentation of the EV.

### Itch Induction (Inside the Scanner)

Average itch intensity during the presentation of the EV in the scanner also significantly differed from the average itch intensity during the CV [*t*(10) = -6,410; *p* ≤ 0.001]. The mean itch intensity during the presentation of the CV was 1.05 ± 1.29, while it was 3.98 ± 2.27 during the presentation of the EV. Itch intensities during the CV and EV are illustrated in **Figure 2**. All participants reported an itch increase of at least 0.75 inside the scanner [range: 0.75–6; measured with a visual analog scale ranging from 0 (no itch) to 10 (worst itch imaginable)].

### Brain Areas Activated under Contagious Itch in Patients with AD

**FIGURE 3:**
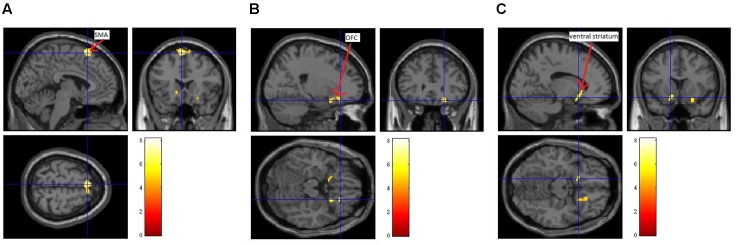
Brain activation during CI in patients with atopic dermatitis. A difference between brain activation during the control condition (presentation of a neutral video) and the presentation of the EV was identified within the supplementary motor area (SMA; **A**), the right orbitofrontal cortex (OFC; **B**) and the left ventral striatum **(C)**.

Three brain regions showed a significant activation [uncorrected *p* < 0.001; cluster size (*k*) > 50] during the presentation of the itch inducing EV in comparison to the baseline condition (presentation of the CV). The presentation of the itch inducing video led to an activation of the SMA, the left ventral striatum and the right OFC (**Figure 3** and **Table 1**).

**Table 1:** Activation during contagious itch (CI).

	EV > CV			
	
	MNI coordinate			*Z*-score
		
Brain regions	*x*	*y*	*z*	
Left SMA	-4	20	64	4.28
Right SMA	8	22	64	3.51
Right OFC	22	30	-18	3.95
	22	14	-18	3.57
Left ventral striatum	-14	14	-14	3.82


*SMA, supplementary motor area; OFC, orbitofrontal cortex.*

### Correlations between Brain Activation and Psychophysical Data

**FIGURE 4:**
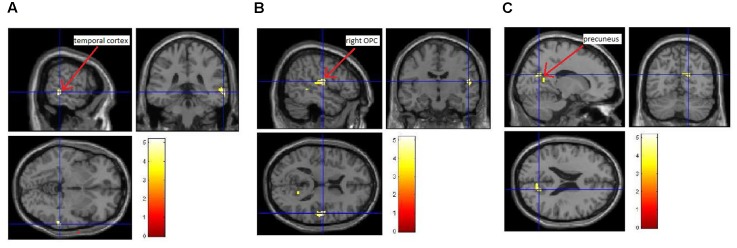
Correlation between induced itch and induced brain activity. We observed a trend of positive correlation (uncorrected *p* < 0.005; *k* > 50) between the increase in itch intensity and the increase in brain activity of the temporal cortex **(A)**, the right pOPC **(B)**, and the (pre-) cuneus **(C)**.

We also investigated whether the increase in itch intensity was significantly correlated to an increase in brain activity. Here, we found that an increase in average itch intensity due to the EV was by trend positively correlated to an activation of the right temporal cortex, the right posterior opercular cortex (pOPC) and the (pre)cuneus (**Figure 4** and **Table 2**; uncorrected *p* < 0.005; *k* > 50).

**Table 2:** Correlation between induced itch and the activation of brain regions during CI.

	EV > CV			
	
	MNI coordinate			*Z*-score
		
Brain regions	*x*	*y*	*z*	
Temporal cortex	64	-36	-4	3.34
Right pOPC	52	-12	14	3.26
	56	-20	12	3.12
(Pre-)Cuneus	16	-64	22	2.84
	16	-54	12	2.81
	8	-68	22	2.70


## Discussion

This study provided an insight into brain processing of CI in AD patients. Knowledge of brain processing of CI in AD may enable to identify target brain regions for treatments which can help AD patients when they are confronted with itch-related stimuli in daily life. Similar to a previous psychophysiological study (Papoiu et al., 2011b) CI was induced by the presentation of an EV showing other persons scratching. As expected, the presentation of this video led to a significant increase in itch and scratching compared to a CV showing the same persons sitting idly.

We found that activity in the SMA, left ventral striatum, and right OFC significantly increased during the presentation of the EV compared to the presentation of the CV. Thus, these regions seem to be crucial for the experience of CI in AD patients. The SMA, ventral striatum, and OFC are involved in the fronto-striatal circuit. This circuit plays an important role for motivation, craving, motor control and preparation, and decision-making (Fried et al., 1991; Krakauer and Ghez, 2000; Smolke et al., 2006; Plassmann et al., 2010; Harsay et al., 2011). In itch perception in particular, this circuit is considered to be associated with the desire to scratch (Mochizuki and Kakigi, 2015). Interestingly, the activation of this circuit was not observed in a previous fMRI study that used a very similar design as ours to investigate brain processing of CI in *healthy subjects* (Holle et al., 2012). A possible explanation for the differences between the two studies is that the fronto-striatal circuit responds excessively to visual itch-related stimuli in AD-patients, which in the following facilitates itch induction and scratching in these patients. This possibility is supported by previous brain imaging studies which found an over-activity in the SMA and the basal ganglia during itch or scratching in chronic itch patients compared to healthy controls (Schneider et al., 2008; Ishiuji et al., 2009; Mochizuki et al., 2015). Therefore, in our point of view, this circuit might be a reasonable target region for non-invasive treatments in AD patients to help them to prevent scratching in itch inducing situations. Interventions that could have an antipruritic effect when being confronted with itch-related stimuli have recently been outlined (Mochizuki et al., 2017): We described that repetitive Transcranial Magnetic Stimulation (rTMS) and transcranial Direct Current Stimulation (tDCS) could be beneficial for patients suffering from chronic itch. Indeed, it has already been shown that tDCS had positive effects in a patient with chronic itch due to neuropathy (Knotkova et al., 2013). In addition, psychological interventions already shown to be effective to lower baseline itch in AD patients (Evers et al., 2009; Bae et al., 2012) might also affect the activity of the fronto-striatal network during CI. We believe that cognitive behavioral therapies including relaxation- and/or habit reversal trainings as well as cognitive restructuring have the potential to alter the patients’ attitude toward visual itch cues, which might then go along with a lower motivation to scratch resulting in a lower brain activity in the fronto-striatal circuit when being confronted with itch-related stimuli. Randomized controlled trials including fMRI measurements before and after treatment are thus needed to prove the effects of different non-invasive interventions on brain activity during the presentation of visual itch cues.

Another interesting finding was that visually induced itch was by trend positively associated with activity in the (pre-) cuneus, pOPC and the temporal cortex. The precuneus and temporal cortex are brain areas that play a role in (episodic) memory retrieval (Cavanna and Trimble, 2006; Palombo et al., 2016). It is possible that seeing somebody else scratching activates the patients’ memories regarding their own experiences with itch-related stimuli, which might then affect the pOPC as a key region of somatosensory processing. It has been demonstrated that spontaneous activation or electrical stimulation of this region can generate pain via the activation of brain network associated with pain (Garcia-Larrea, 2012). Considering similarity of brain network between itch and pain (Ständer and Schmelz, 2006; Yosipovitch et al., 2007), it may be possible that memory retrieval depicted by the activation of the precuneus and temporal cortex activates the pOPC, which induces the significant activation of the fronto-striatal circuit in AD-patients.

Moreover, we would like to stress the role of the precuneus for the experience of itch in *AD patients*. This region has been shown to be involved in the processing of itch transmitted by histaminergic and non-histaminergic pathways (Mochizuki et al., 2009; Papoiu et al., 2012), and to be significantly more activated during itch processing in AD patients compared to healthy controls (Ishiuji et al., 2009). Interestingly, this region has been shown to be not involved in pain processing (Apkarian et al., 2005; Yosipovitch and Mochizuki, 2015). Instead, an activation of the precuneus has been linked to empathy (Farrow et al., 2001). We speculate that in the context of CI an activation of the precuneus could be seen as a sign of a high ability of taking the perspective of others. In future studies it would therefore be interesting to test the hypothesis that very empathetic patients show more intense itch and more scratching behavior during CI and that this more profound response goes along with more activation in the precuneus.

There are some limitations to this study, which need to be addressed. First, one might argue that we cannot distinguish between whether the activation of the brain regions that we found is “CI specific” or rather a response to the observation of actions, because videos also differed in that way that people in the EV moved while they were sitting idly in the CV. However, previous brain imaging studies investigating the cerebral processing of action observation have not observed the activation of the regions that we observed. Instead they have commonly observed the activation of other brain regions such as the dorsolateral prefrontal cortex (DLPFC), premotor cortex (PM), and parietal cortex (Buccino et al., 2004a,b; Calvo-Merino et al., 2006; Iacoboni and Dapretto, 2006). The results of these previous studies in combination with our profound increase in itch/scratching seen in the psychophysical data strengthen our confidence that we actually measured CI related brain activity and not brain activity that occurred due to the observation of actions. As a second limitation, our sample size was rather small with *n* = 11 patients and data of a healthy control group are not included in this study. However, the purpose of the present study was to investigate which parts of the brain are activated in patients with chronic itch while they view scratching behavior in others, and not to identify brain regions responsible for the robustness of CI seen in AD patients in comparison to healthy subjects. Therefore, our study should be regarded as a pilot study. A further fMRI study including a larger sample of patients and healthy participants could narrow down candidate brain regions responsible for the augmented CI seen in AD patients. This may finally lead to the identification of target regions for non-invasive treatments of CI in patients with AD (Mochizuki et al., 2017). Third, also confounding factors like the patients’ personality and the expectations regarding the upcoming itch stimuli that have recently been shown to be associated with the patients’ response to audiovisual itch cues (Schut et al., 2014, 2016) should be taken into consideration in future studies with larger sample sizes.

Despite these limitations, this study was the first pilot study to elucidate mechanisms of CI in the brain of patients with atopic eczema suffering from chronic itch. Future studies that would examine whether similar mechanisms occur in other types of chronic itch would be of major interest.

## Author Contributions

CS overall conceptualization of the study, data collection, statistical analysis, participation in analyses of the MRI data, and writing the paper. HM contributions to the conceptual design, statistical analysis, supervision of data collection, analyses of the MRI data, and writing the paper. SG analysis of scratching behavior, data input. AL analysis of scratching behavior. CC supervision of MRI data collection, participation in analyses of the MRI data, discussion of results. FM supervision of MRI data collection, participation in analyses of the MRI data, discussion of results. UG participation in overall conceptualization of the study. JK participation in overall conceptualization of the study, participation in statistical analysis of psychophysical data, discussion of results. GY participation in overall conceptualization of the study, participation in recruitment of participants, discussion of results, and writing the paper. All authors read and approved the manuscript.

## Footnotes

1 http://www.fil.ion.ucl.ac.uk/

2 https://cfn.upenn.edu/~zewang/ASLtbx.php
